# Co-evolution as Tool for Diversifying Flavor and Aroma Profiles of Wines

**DOI:** 10.3389/fmicb.2018.00910

**Published:** 2018-05-07

**Authors:** Peter Morrison-Whittle, Soon A. Lee, Bruno Fedrizzi, Matthew R. Goddard

**Affiliations:** ^1^School of Biological Sciences, University of Auckland, Auckland, New Zealand; ^2^School of Chemical Sciences, University of Auckland, Auckland, New Zealand; ^3^The School of Life Sciences, University of Lincoln, Lincoln, United Kingdom

**Keywords:** wine yeast, co-evolution, metabolite analysis, microbial interactions, co-culture

## Abstract

The products of microbial metabolism form an integral part of human industry and have been shaped by evolutionary processes, accidentally and deliberately, for thousands of years. In the production of wine, a great many flavor and aroma compounds are produced by yeast species and are the targets of research for commercial breeding programs. Here we demonstrate how co-evolution with multiple species can generate novel interactions through serial co-culture in grape juice. We find that after ~65 generations, co-evolved strains and strains evolved independently show significantly different growth aspects and exhibit significantly different metabolite profiles. We show significant impact of co-evolution of *Candida glabrata* and *Pichia kudriavzevii* on the production of metabolites that affect the flavor and aroma of experimental wines. While co-evolved strains do exhibit novel interactions that affect the reproductive success of interacting species, we found no evidence of cross-feeding behavior. Our findings yield promising avenues for developing commercial yeast strains by using co-evolution to diversify the metabolic output of target species without relying on genetic modification or breeding technologies. Such approaches open up exciting new possibilities for harnessing microbial co-evolution in areas of agriculture and food related research generally.

## 1. Introduction

For thousands of years humans have benefited from the products of microbial metabolism as they form the basis of all fermented foods and beverages (Blandino et al., 2003; Hutkins, 2007). In wine production, yeast species metabolize sugars and other compounds in grape juice and convert these into alcohol and a vast array of flavor and aroma compounds (Pretorius, 2000; Ciani et al., 2010). It is beneficial to have some control over the balance of desirable metabolites in the final wine, as this underpins the quality and value of finished wines. A large fraction of wine metabolites are produced by a variety of yeasts found naturally associated with grapes and their ferments, and yeast metabolism has been the subject of intensive research for many years (Pretorius, 2000; Ciani et al., 2010; Knight et al., 2015). Traditionally, harnessing desirable yeast metabolites has been achieved through breeding programs or by genetic modification of the main fermentative species: *Saccharomyces cerevisiae*. Over the last few years however, researchers have begun to explore the possibilities of altering the balance of flavor and aroma metabolites by inoculating more than one species of yeast into commercial ferments (Anfang et al., 2009; Ciani et al., 2010). Here we take this a step further and demonstrate the ability to harness microbial interactions using co-evolution as a means of diversifying and altering the metabolism of yeast species.

Microbial metabolism is influenced by a number of factors and classically, is understood to be largely a product how a microbe's genome interacts with its physical and chemical environment. Just as a microbe's genome will be subject to evolutionary change across multiple generations, so too will the manifestation of genome evolution on metabolism. A number of studies have demonstrated that microbial metabolism can significantly shift over a number of generations when grown consistently in controlled conditions (e.g., Fong et al., 2005; Gresham et al., 2008; Behe, 2010; Padfield et al., 2016). In addition to these adaptive shifts in metabolism in response to novel environments, the presence of other species may also alter the metabolism and evolutionary trajectories of bacterial species (Lawrence et al., 2012; Barraclough, 2015).

Species interactions have a profound effect on the evolution and ecological dynamics of biological species (Cadotte et al., 2008; Harmon et al., 2009; Bassar et al., 2010; Poltak and Cooper, 2011). These interactions may be broadly categorized as: antagonistic (competition, predation, ammensalism, and parasitism); neutral (such as commensalism); or mutualistic (such as cross-feeding)—as reviewed in West et al. (2007). The origin of these interactions through co-evolution has important consequences for overall metabolic regulation/flux (West et al., 2007). Lawrence et al. (2012) demonstrated the use of co-culture with serial transfers as a means of inducing co-evolution between bacterial species. In doing so, they demonstrated that bacteria independently and co-evolved showed significantly different reproductive success when subsequently co-cultured, that was consistent with evolved mutualistic cross-feeding behavior in co-evolved lines. Furthermore, the authors showed that these novel interactions were associated with significantly different patterns of metabolic regulation in co-evolved species.

In this study, we apply the experimental approach of Lawrence et al. (2012) to evaluate the evolution of novel microbial interactions between microbial eukaryotes: the grape and wine ferment associated yeasts *Candida glabrata* and *Pichia kudriavzevii*. We go on to quantify the impact of co-evolution on the production of 38 commercially important flavor and aroma compounds produced during experimental ferments with *S. cerevisiae*.

## 2. Methods

### 2.1. Selection of fungal species

Initially 96 vineyard derived non-*Saccharomyces* isolates from our culture collection were grown in commercially harvested Sauvignon Blanc juice deriving from Marlborough, New Zealand. The SO_2_ concentration of this juice, hereafter referred to as “juice A”, was adjusted to 20 mg/L. Each non-*Saccharomyces* isolate was added to 200 μL of juice and incubated for 24 h. Isolates that grew readily (as measured by optical density) were then grown on yeast-extract peptone dextrose (YPD) agar to determine colony morphology. For ease of identification in co-cultures, isolates of different colony morphology were paired. All combinations of isolate pairs were then co-inoculated separately into juice A and incubated for either 24, 48, or 72 h. Ultimately, the final isolate pairs selected from all co-culture combinations were those that: (1) grew quickly in juice A over a 24-h period; and (2) grew at similar rates, yielding approximately equal numbers of colonies after spread-plating co-culture aliquots on YPD agar. Of these plated isolates, two colony phenotypes predominated, and were *Candida glabrata* and *Pichia kudriavzevii*. The identity of these isolates was confirmed by sequence homology of PCR amplicons at the D1/D2 26S rDNA locus using NL1 and NL4 primers (Kurtzman and Robnett, 2003; Romanelli et al., 2010).

### 2.2. Serial transfers

**Figure 1:**
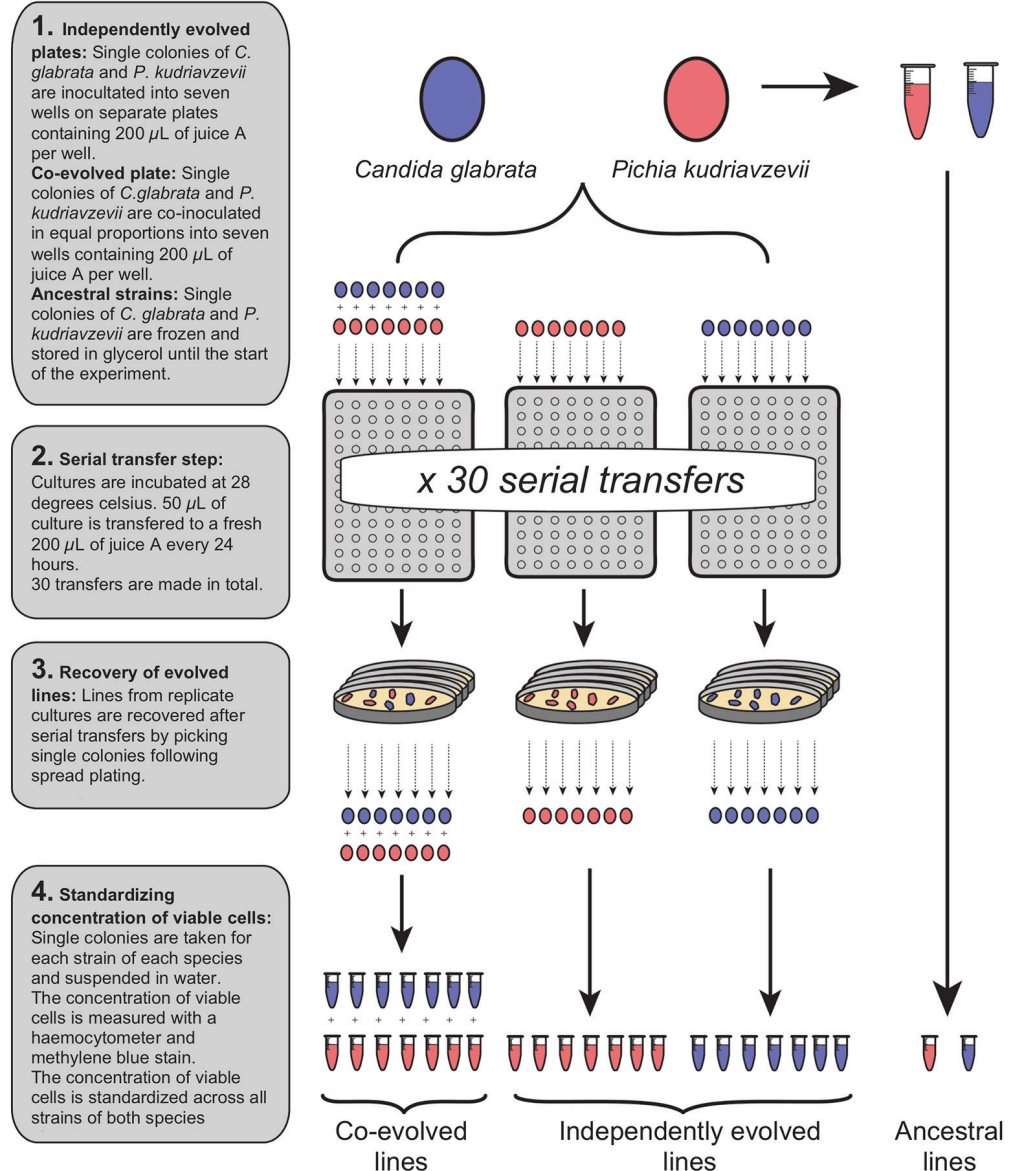
Generation of independently evolved and co-evolved yeast lines from a single ancestral colony by serial transfer in fresh juice.

Two experimental groups were initiated: “independently evolved” and “co-evolved”. All independently evolved and co-evolved isolates of *C. glabrata* and *P. kudriavzevii* derived from a single colony of each species, and the ancestral isolates were stored in suspended animation at −80°C. The generation of all experimental strains are shown in Figure 1. Independently evolved lines were prepared by suspending *C. glabrata* and *P. kudriavzevii* in distilled water at equivalent optical densities, and 50 μL inoculated into seven wells (biological replicates) of 96-deepwell plates containing 200 μL of juice A for each. Distilled water was added to 200 μL of juice A in one well to act as a negative control for each plate.

Co-evolved lines were prepared by thorough vortex-mixing a 50:50 mix of the two species (See Figure 1). From this combined suspension, 50 μL was inoculated into 200 μL of juice A in seven wells of a 96-deepwell plate, and distilled water added to appropriate wells to act as a negative control. The two plates containing independently evolved lines and the single plate containing co-evolved lines were incubated at 28°C for 24 h. After 24 h, the contents of each well in each plate were mixed by pipetting, and 50 μL of each culture was transferred to 200 μL of fresh juice A, and incubated at 28°C for 24 h. This transfer procedure to fresh juice was repeated a total of 30 times to continuously grow independently evolved and co-evolved lines for ~65 generations. Independently evolved and co-evolved isolates of *C. glabrata* and *P. kudriavzevii* were recovered after serial transfer by spread plating on YPD, from which single colonies were isolated and stored in15% (v/v) glycerol at −80°C (See Figure 1).

### 2.3. Growth media

While the independently evolved and co-evolved lines of *C. glabrata* and *P. kudriavzevii* were evolved in juice A, downstream growth and metabolite assays were also carried out in a second juice B. Juice B was prepared as a blend from a number of other Sauvignon Blanc juice stocks donated by various commercial wineries. Conducting all analyses in two juices allowed us to test whether any significant differences between independently evolved and co-evolved lines were specific to the environment (juice) in which they evolved, or whether any evolved interactions were also expressed in different environments (juice chemistries). For both juices, 10 L of frozen juice was thawed prior to inoculation and sterilized at room temperature overnight using dimethyl dicarbonate (DMDC) in 25 L carboys. Each juice was mixed thoroughly and 200mL was dispensed into sterilized 250 mL flasks with one-way airlocks 24 h prior to inoculation with *C. glabrata* and *P. kudriavzevii* strains.

### 2.4. Flask ferments

**Figure 2:**
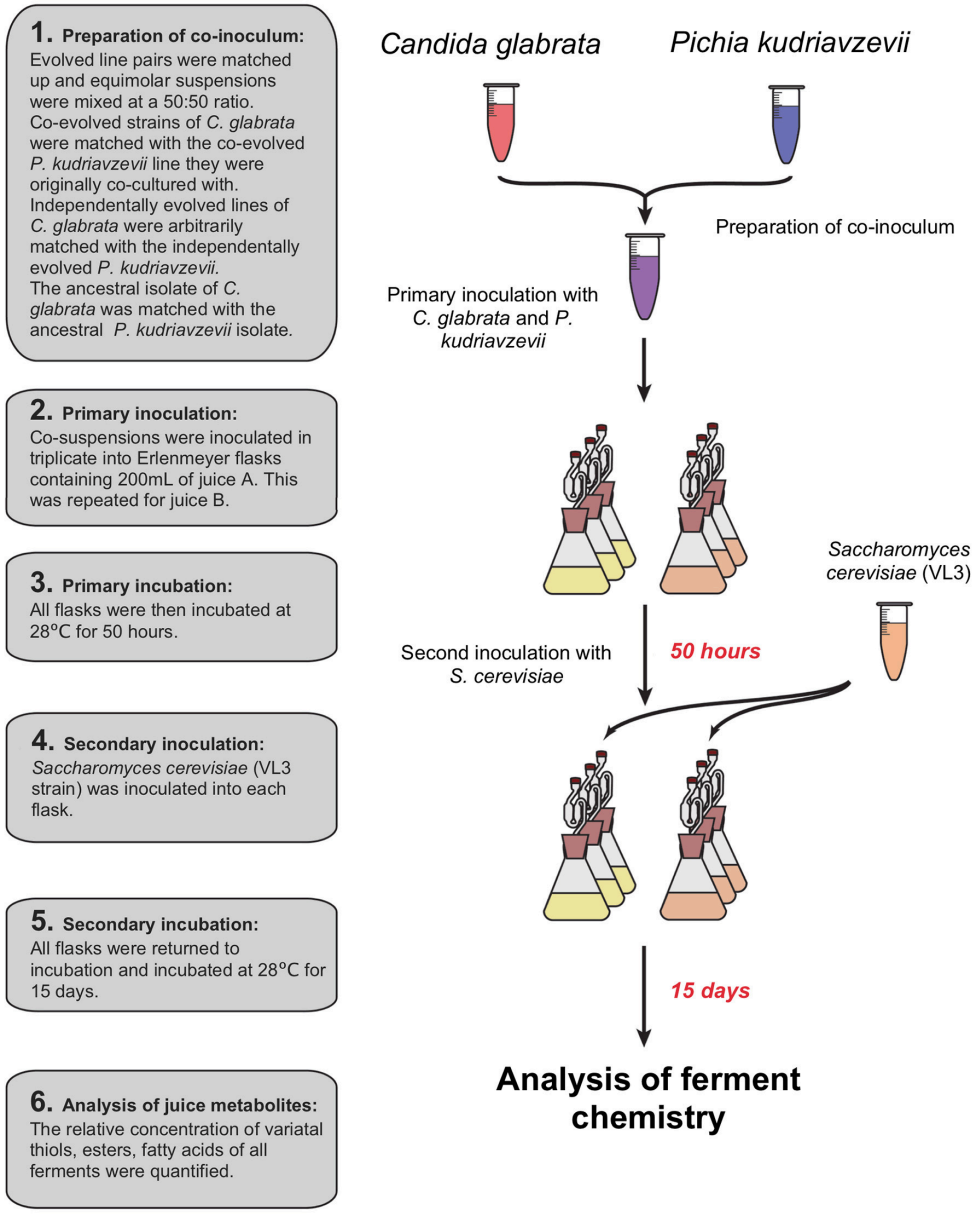
Sequential inoculation of experimental ferments: primary inoculation of *C. glabrata* and *P. kudriavzevii* pairs then a secondary inoculation of *S. cerevisiae* after 50 h.

Prior to inoculation of juices A and B, *C. glabrata* and *P. kudriavzevii* were recovered from glycerol storage by growth in YPD, and each sample was then transferred to 50 mL Falcon tubes and pelleted at 3,000 g for 5 min. The resulting pellets were re-suspended in 10 mL of distilled water and transferred to fresh 15 mL Falcon tubes. The concentration of viable cells was enumerated using a haemocytometer with methylene blue staining solution. The concentration of viable cells was standardized to the sample with the lowest cell concentration. *C. glabrata* and *P. kudriavzevii* co-evolved strains were re-paired with their respective partner. Independently evolved strains of *C. glabrata* and *P. kudriavzevii* were paired arbitrarily. Flasks were co-inoculated by inoculating 1 mL of both *C. glabrata* and *P. kudriavzevii*, resulting in a final concentration of 2.52 × 10^5^ cells mL^−1^ for both species (see Figure 2). All inoculated juice was then incubated for 50 h at 28°C after which all were inoculated with the same VL3 commercial strain of *S. cerevisiae* to a final concentration of 2.38 × 10^3^ cells mL^−1^ to emulate a commercial situation and ferment to dryness. Flasks were incubated for 15 days at 28°C. After fermentation, cells were pelleted by centrifugation at 3,000 g for 5 min after which the supernatant was decanted and stored at −20°C for downstream juice metabolite analysis.

### 2.5. Bioscreen C™ growth assays

Relative fitness was estimated by maximum growth rate (V_*max*_), lagtime, and cell-densities at 12, 24, and 48 h using Bioscreen C™ spectrophotometer/incubator. Maximum growth rate is defined as the maximum change in optical absorbance (wideband filter 420–580 nm) over a sliding 10 hr window; lagtime was defined as the time until a culture reached V_*max*_, and cell density was approximated by optical absorbance. Each strain was grown from frozen glycerol stocks in liquid YPD for 24 h prior to analysis. The concentration of viable cells was enumerated using methylene blue stain and all samples were standardized to the sample of the lowest concentration of viable cells. 15 μL of each strain suspension was added to separate 100-well Bioscreen plates (300 μL capacity) containing 185 μL of juice A and B separately. Each strain was inoculated into five technical replicates per treatment, producing a final concentration of 2.9 × 10^3^ viable cells per well.

### 2.6. Metabolite analysis of co-inoculated ferments

To evaluate metabolic output, the relative concentrations of two varietal thiols, fifteen esters, six higher alcohols, four C6 compounds, six terpenes, and five fatty acids of all ferments were quantified following the method described in Knight et al. (2015). Varietal thiols (3MH, 3MHA) were quantified using an ethyl propiolate derivatization and analyzed on an Agilent 6890N gas chromatograph (Santa Clara, CA, USA) equipped with a 7683B automatic liquid sampler, a G2614A autosampler and a 593 mass selective detector as outlined in Herbst-Johnstone et al. (2013b). Esters, alcohols, C6 compounds, terpenes, and fatty acids were quantified simultaneously using a HS-SPME/GC-MS method outlined in Herbst-Johnstone et al. (2013a). Raw data was transformed with GCMSD Translator and peak integration was performed using MS Quantitative Analysis, both part of the Agilent MassHunter Workstation Software (Version B.04.00, Agilent Technologies).

### 2.7. Species identification/contamination controls

DNA from single colonies was extracted using Zymo Soil DNA extraction kits (Irvine, CA, USA), and species identity confirmed through Sanger sequencing of the D1/D2 region of the 26S rDNA using NL1 and NL4 fungal primers (Kurtzman and Robnett, 2003). One isolate recovered from the co-evolved *C. glabrata* serial-transfer plate could not be amplified using fungal primers and appeared to be a bacterial contaminant. All ferment and Bioscreen samples that contained this contaminant were excluded from all analysis.

### 2.8. Statistical analysis

To test whether independently evolved and co-evolved lines had significantly different growth rates and cell-densities, separate one-way full factorial ANOVAs were conducted for each juice, testing: maximum growth rate (V_*max*_); lagtime; and cell densities at 12, 24, and 48 h after inoculation. To test whether the metabolic profiles of independently evolved and co-evolved lines significantly differed from each other, we implemented two-way full factorial permutational multivariate ANOVA (permanova) of Jaccard dissimilarities between metabolite profiles. Separate tests were conducted for: esters, fatty acids, terpenes, C6 compounds, and all metabolites combined.

## 3. Results

### 3.1. Relative fitness of evolved *C. glabrata* and *P. kudriavzevii*

**Figure 3:**
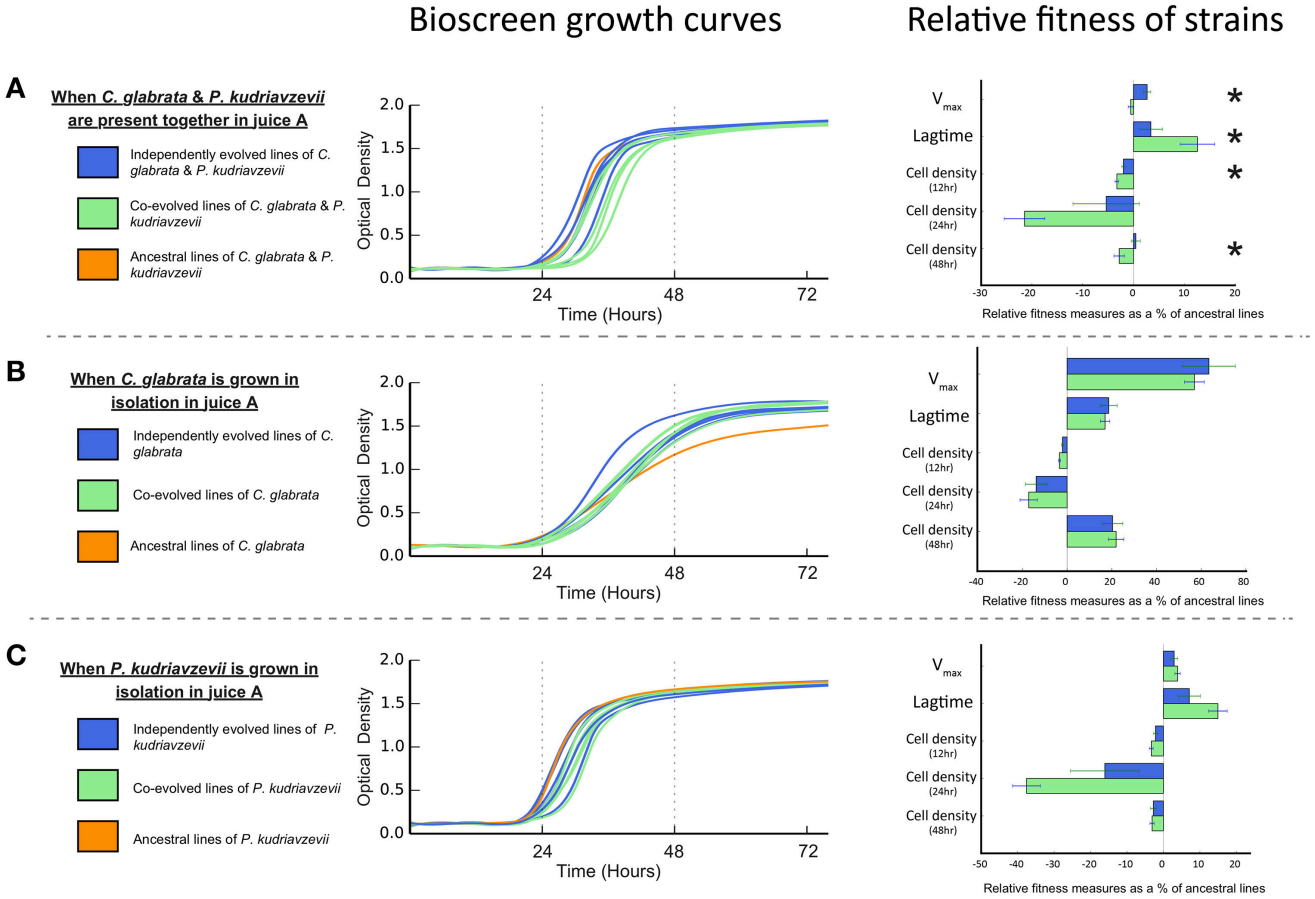
Bioscreen growth curves and relative fitness measures in juice A of co-evolved, independently evolved, and ancestral lines of **(A)** *C. glabrata* and *P. kudriavzevii* when grown together. **(B)** *C. glabrata* when grown in isolation. **(C)** *P. kudriavzevii* when grown in isolation. Relative fitness measures—V_*max*_, lagtime, and cell densities—are expressed as the proportional difference between evolved lines and the ancestral line. Significant differences between co-evolved and independently evolved strains are denoted by “^*^”.

The evolution of interactions between microbes may be manifest in differential reproductive success (or fitness). Antagonistic interactions are predicted to lower the net reproductive success when in co-culture (Lawrence et al., 2012). Conversely, mutualistic interactions, such as cross-feeding, are predicted to increase the net reproductive co-culture success (Lawrence et al., 2012). Bioscreen analyses show co-cultures of co-evolved *C. glabrata* and *P. kudriavzevii* pairings have significantly lower net V_*max*_ than co-cultures of individually evolved strains [*F*_(1, 9)_ = 13.266, *P* = 0.005382; see Figure 3A]. Moreover, the lagtime of co-evolved co-cultures was significantly greater than individually evolved co-cultures [*F*_(1, 9)_ = 5.2517, *P* = 0.04765], which reached peak growth rate roughly 2 h sooner. This difference in net growth rate between co-evolved and individually evolved strain pairs resulted in co-cultures having lower cell densities after 12 h [*F*_(1, 9)_ = 7.2366, *P* = 0.02479] and 48 h [*F*_(1, 9)_ = 5.7289, *P* = 0.04032], but this difference was not significant at 24 h [*F*_(1, 9)_ = 3.9378, *P* = 0.0785].

Lawrence et al. (2012) found that cross-feeding behavior between co-evolved bacterial species represented an adaptive trade-off, as co-evolved strains had significantly lower fitness than independently evolved equivalents when grown in isolation. To test whether such an adaptive trade-off was apparent in these microbial eukaryotes, we measured the growth rates and cell densities of both individually evolved and co-evolved *C. glabrata* and *P. kudriavzevii* when grown in isolation. Co-evolved strains of *C. glabrata* were just as fit as individually evolved lines (as shown in Figure 3B) when grown in isolation, as there was no significant difference in V_*max*_ or lagtime [V_*max*_: *F*_(1, 9)_ = 0.2244, *P* = 0.647; lagtime: *F*_(1, 9)_ = 0.125, *P* = 0.7318. We found no significant difference between the cell densities of co-evolved and individually evolved *C. glabrata* at 12, 24, and 48 h [12 h : *F*_(1, 9)_ = 3.5403, *P* = 0.09258; 24 h : *F*_(1, 9)_ = 0.2687, *P* = 0.6167; 48 h: *F*_(1, 9)_ = 0.0712, *P* = 0.7956]. Co-evolved *P. kudriavzevii* were equally as fit as individually evolved lines (see Figure 3C), with no significantly difference in V_*max*_ [*F*_(1, 9)_ = 0.5513, *P* = 0.4767] or lagtime [*F*_(1, 9)_ = 3.6597, *P* = 0.08803]. As was observed with *C. glabrata*, co-evolved and individually evolved *P. kudriavzevii* did not have significantly different cell-densities at 12, 24, or 48 h [12 h: *F*_(1, 9)_ = 2.2205, *P* = 0.1704; 24 h: *F*_(1, 9)_ = 3.808, *P* = 0.08278; 48 h: *F*_(1, 9)_ = 0.1226, *P* = 0.7343]. The fact that cell densities are equivalent between individually and co-evolved lines for each species shows that these populations have expanded to the same extents at each cycle. Thus, both treatments (individually and co-evolved) experienced approximately the same number of generations across the experiment for *C. glabrata* and *P. kudriavzevii*.

### 3.2. The relative fitness of evolved strains across juices

Co-evolved lines displayed decreased fitness compared to individually evolved lines for some fitness components when subsequently co-cultured in a different juice (see Figures S1, S5, S6). Co-evolved lines had significantly longer lagtimes than individually evolved lines [*F*_(1, 9)_ = 11.729, *P* = 0.007571], but they did not have significantly different V_*max*_ [*F*_(1, 9)_ = 3.1321, *P* = 0.1105] in juice B. Co-evolved lines showed significantly lower cell densities than individually evolved lines at 12 h [V_*max*_: *F*_(1, 9)_ = 9.3651, *P* = 0.01357], but not at 24 or 48-h [24 h: *F*_(1, 9)_ = 0.7227, *P* = 0.4173; 48 h: *F*_(1, 9)_ = 4.5186, *P* = 0.06246] in juice B.

There was no significant difference in growth rate or cell density (see Figure S1B) between co-evolved and individually evolved lines of *C. glabrata* in juice B. However, co-evolved *P. kudriavzevii* were less fit than individually evolved in juice B for some fitness components (see Figure S1C): co-evolved lines showed no significant difference in V_*max*_ or lagtime [V_*max*_: *F*_(1, 9)_ = 0.903, *P* = 0.3668; lagtime: *F*_(1, 9)_ = 0.7421, *P* = 0.4113], but did show significantly lower cell densities than individually evolved lines in Juice B at 12 h [*F*_(1, 9)_ = 19.428, *P* = 0.001701], but not at 24 and 48 h [24 h: *F*_(1, 9)_ = 1.1411, *P* = 0.3132; 48 h: *F*_(1, 9)_ = 1.1933, *P* = 0.303].

### 3.3. The evolution of metabolite profiles

We quantified the relative abundance of 38 metabolites in wine fermented by the variously treated *C. glabrata* and *P. kudriavzevii* lines, along with VL3. Both juice-type and evolution status significantly affected the overall metabolite profiles as indicated by two-way permanova analysis of Jaccard dissimilarities (Table 1), but there was no significant interaction between these (*R*^2^ = 0.0128, *P* = 0.6279). This difference persists when broken down into major metabolic groups, but with varying levels of significance: esters (Juice: *R*^2^ = 0.2210, *P* = 0.0121; strain status: *R*^2^ = 0.1508, *P* = 0.0334); C6 compounds (Juice: *R*^2^ = 0.7839, P < 0.0001; strain status: *R*^2^ = 0.0375, *P* = 0.0309); and terpenes (Juice: *R*^2^ = 0.5273, P < 0.0001; strain status: *R*^2^ = 0.0882, *P* = 0.0203)—see Table S1. Of all classes, co-evolution had the greatest effect on ester profiles, where co-evolution induced approximately two-thirds the magnitude of the effect of juice in determining changes of esters profiles (Esters—effect of juice: *R*^2^ = 0.221; effect of evolution status: *R*^2^ = 0.151).

**Table 1:** Results of Permutation ANOVA of Jaccard dissimilarities between overall metabolite profiles using independently evolved and co-evolved lines across two juices (9,999 permutations).

**Effect**	***df***	***SS***	***MS***	***F*_*pseudo*_**	***R*^2^**	***P***
Juice	1	0.62054	0.62054	14.4233	0.38460	0.0005
Culture status	1	0.19789	0.19789	4.5995	0.12265	0.0222
Interaction	1	0.02063	0.02063	0.4795	0.01279	0.6279
Residuals	18	0.77442	0.04302		0.47997	
Total	21	1.61347			1.00000	

*df, degrees of freedom; SS, sum-of-squares; MS, mean sum-of-squares; F_pseudo_, pseudo F-statistic; R^2^, R-squared value; P, p-value*.

**Figure 4:**
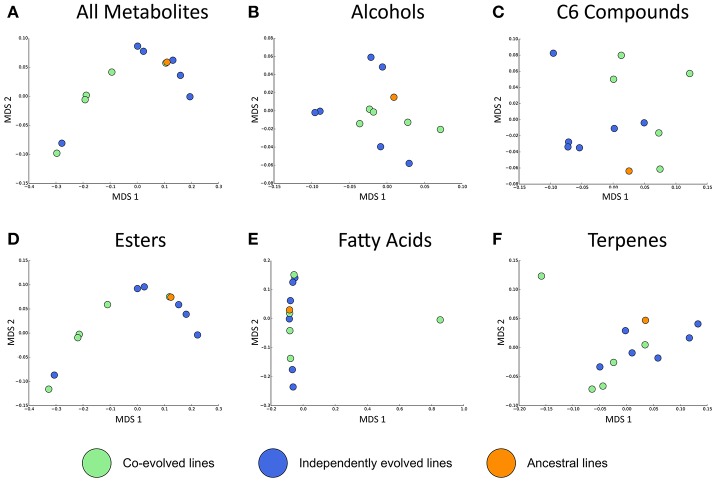
Jaccard dissimilarities of metabolite profiles—averaged across technical replicates—for **(A)** All metabolites, **(B)** Higher alcohols, **(C)** C6 compounds, **(D)** Esters, **(E)** Fatty acids, **(F)** Terpenes in Juice A.

**Figure 5:**
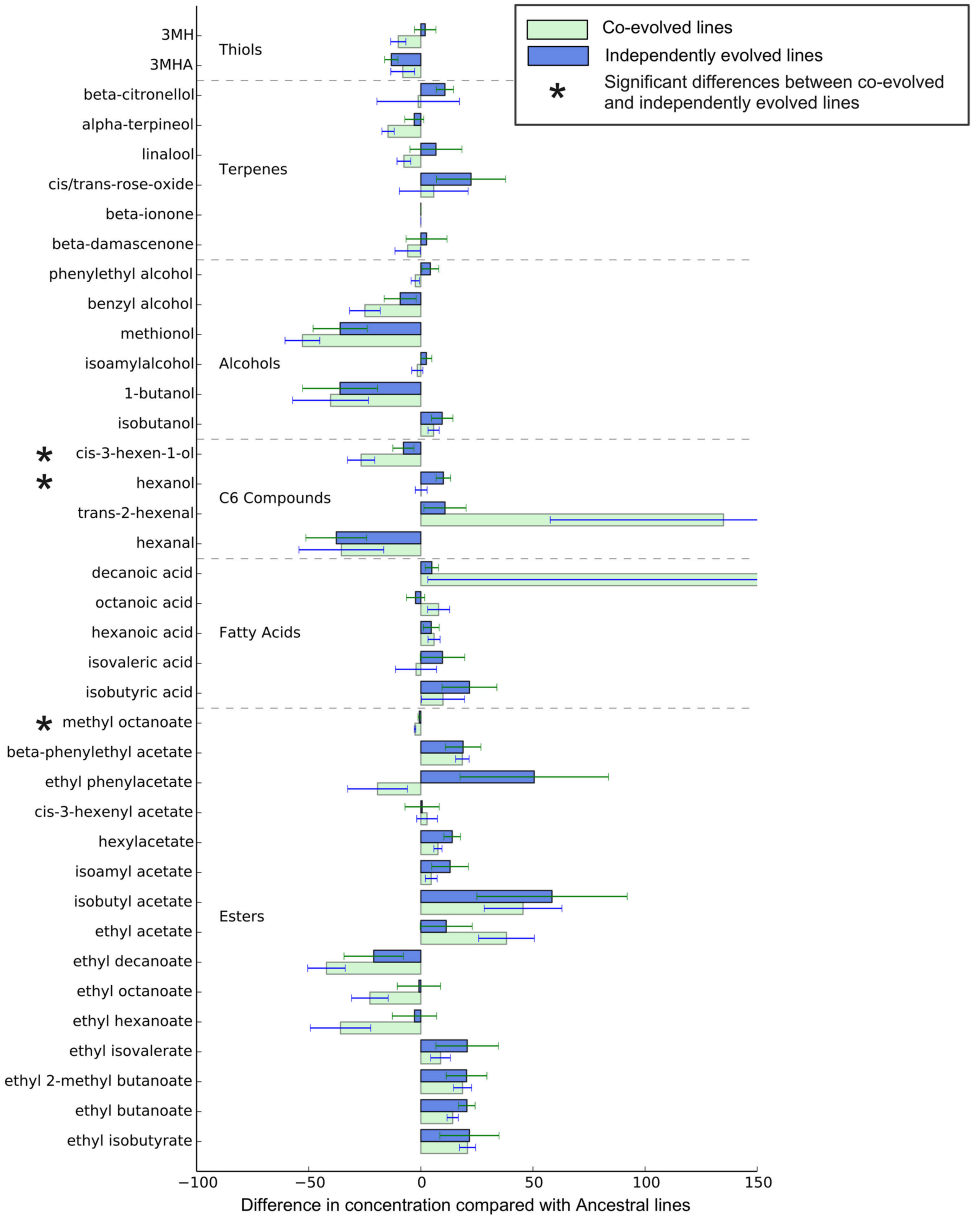
Relative metabolite concentrations of co-evolved and independently evolved lines in Juice A—the juice they were evolved in. The concentrations of metabolites are the proportional difference in concentration compared to the ancestral line.

Overall, the effect of environment (juice) explains three times the variation than whether isolates were co-evolved or individually evolved, but the effect of evolution status is significant, and these are displayed in multidimensional scaling plots (see Figure 4 and Figures S2–S4). Evolution status significantly impacted the metabolite profiles overall, and analyses of individual metabolite concentrations indicate a number of compounds which drive this difference, particularly increases in trans-2-hexenal and decanoic acid, and decreases in 3 MH and ethyl phenylacetate. The relative abundances of each compound in derived lines compared to ancestral lines is shown in (Figure 5 and Figure S7).

## 4. Discussion

We found that after ~65 generations of co-culture in Sauvignon Blanc juice, *C. glabrata* or *P. kudriavzevii* appear to have co-evolved, and that this co-evolution has significantly shifted the balance and composition of many of the flavor and aroma compounds we quantified. This study demonstrates the use of co-evolution as a means of diversifying the metabolic products of commercially important microbes. To the best of our knowledge, this is the first time that the evolution of microbial interactions in the lab has been shown to significantly modify the metabolite profiles of experimental wine ferments.

Contrary to the findings of Lawrence et al. (2012) we did not find evidence of the evolution of cross-feeding indicating the evolution of mutualistic interactions. Instead we see co-evolved strains of *C. glabrata* and *P. kudriavzevii* display lower V_*max*_ and cell densities than independently evolved strains. The reduced fitness of the co-evolved strains when grown together is consistent with antagonistic interactions between species that appear absent in independently evolved equivalents. When interactions between microbes are antagonistic, chemical energy available for reproduction is reduced by the metabolic costs of stress responses elicited by other microbes or on producing metabolites that reduces the reproductive success of other microbes.

One important consideration of utilizing co-evolution to alter microbial metabolism is generation time. As the number of generations increases, so does the likelihood that the phenotype of different evolutionary lines will diverge from one another. It is possible that the apparently antagonistic interaction between co-evolved *C. glabrata* and *P. kudriavzevii* may not represent a stable evolutionary state, and may intensify or change entirely given more generation time. A number of studies of experimental co-cultures have reported that the nature of microbial interactions do change over time (Poltak and Cooper, 2011; Andrade-Domínguez et al., 2014); some become increasingly mutualistic, others increasingly antagonistic. This phenotypic variation through time further increases the pool of yeast phenotypes from which strains can be selected and bred from as transitional phenotypes can be archived in glycerol storage.

It is important to note that this experimental design does not resolve whether the up-regulation or down-regulation of any one compound is a result of adaptation to other members of co-culture. Metabolic traits may not be adaptive in themselves but may covary with traits that are through gene linkage (Gould and Lewontin, 1979). Furthermore, our experimental design does not allow us to determine what species is driving the abundance of any one metabolite. For example, it is unclear whether *C. glabrata* or *P. kudriavzevii* directly affect the concentration of sensory compounds (by producing or metabolizing them) or whether they affect them indirectly by altering the metabolism of one or more co-fermenting partners. What this study does show is that the co-evolution of yeast strains naturally present on fruits and their ferments may be employed to manipulate the products of commercial fermentation.

Another key consideration for this study is species number. In this study we report on interactions between two species, but it should be noted that the complexity and nature of microbial interactions can differ dramatically depending on the numbers present in co-culture (Barraclough, 2015; Fiegna et al., 2015). Lawrence et al. (2012) used 4 bacterial species in a simulated community and detected evidence of mutualistic co-evolution. Fiegna et al. (2015) found in experimentally assembled biofilm communities that species interactions evolved to be less negative over time, particularly in diverse communities. It seems reasonable to suggest that the nature and impact of microbial interactions on metabolite profiles may vary depending on the number and types of yeast species used. This complexity greatly enhances the potential for commercial researchers to generate a vast number of possible phenotypes—and subsequently, flavor and aroma profiles—by co-evolving a small number of yeasts in different combinations.

It should also be noted that antagonistic, neutral, or mutualistic microbial interactions do not predict whether the interaction is commercially valuable. The value of any microbial interaction in changing the metabolite profiles of any commercially valuable microbe depends on what metabolite profile is considered desirable. Inducing co-evolution between wine yeasts merely represents a tool for diversifying the metabolite output of prospective yeast species. By diversifying the possible phenotype of yeast species, one can increase the pool from which strains can be selected, bred from, or used directly.

Furthermore, while this study infers that serial co-culture significantly alters microbial metabolism as the result of evolutionary change, we did not quantify any sequence changes in the genomes after serial co-culture beyond Sanger sequencing of a single locus. Here we demonstrate that serial co-culture significantly altered microbial metabolism and that this metabolic variation was heritable and persisted in subsequent generations after the co-culture step. However, as we did not quantify and genetic change, we cannot exclude the possibility that the changes in microbial metabolism are a consequence of epigenetic changes and not changes in genomic sequences, but we can conclude these changes are heritable. We would argue that if natural selection for microbial interaction is driving the formation and maintenance of these genetic and/or epigenetic changes, then the genetic changes in the genome would be predicted given enough generations.

Co-evolution is a powerful mechanism with which researchers can diversify or differentiate the metabolic activity of scientifically and/or commercially important organisms. Interactions between yeasts in commercial ferments, whether coincidental or derived from co-evolution, undoubtedly play a role in shaping the sensory properties of many commercial wines, especially those produced by spontaneous fermentation of harvested grape juice. Fermentative foods represent a powerful model for dissecting processes of microbial community formation (Wolfe and Dutton, 2015). Here we demonstrate the potential for utilizing both biotic and abiotic pressures to diversify the metabolic activity of commercially valuable yeast species. This study provides a tentative insight into the commercial value of microbial co-evolution; the practical applications of controlling wine sensory properties are vast, and elucidating the many mechanisms of evolution opens up exciting new areas of agriculture and food related research generally.

## Author contributions

PM-W conceived and design the overall experimental design, trialed and selected non-*Saccharomyces* yeast isolates, carried out the continuous co-culture design through serial transfers of co-culture to fresh media, extracted DNA from all yeast isolates carried out BioscreenTM analysis of growth rates and cell densities, prepared juices A and B for experimental fermentation, carried out experimental fermentation of juices in 200 ml flasks, prepared resultant wine samples for downstream metabolite analysis, statistically analyzed all data, prepared primary manuscript, and proof-read and prepared final manuscript. SL confirmed the species identity of experimental strains through Sanger sequencing of D1/D2 26S locus amplified by PCR using NL1 and NL4 primers. Soon also quantified the concentrations of variatal thiols, esters, terpenes, C6 compounds, higher alcohols and fatty acids present in wine samples derived from experimental fermentation. BF provided expertise with volatile measurements. MG assisted in the experimental design, and assisted in the preparation of the final manuscript.

### Conflict of interest statement

The authors declare that the research was conducted in the absence of any commercial or financial relationships that could be construed as a potential conflict of interest.
