# Supplementary material for: Co-evolution as Tool for Diversifying Flavor and Aroma Profiles of Wines
Source: Front Microbiol. 2018 May 7;9:910. doi: 10.3389/fmicb.2018.00910 (PMC5949342; doi:10.3389/fmicb.2018.00910)
Supplement: Supplementary file 1 [file Presentation_1.PDF]

Supplementary Material: Co-evolution as tool for diversifying flavour and aroma profiles of wines

1 SUPPLEMENTARY DATA

1.1 Tables

Table S1. Results of Permutation ANOVA of Jaccard dissimilarities between Esters, Fatty acids, C6 compounds, Higher alcohols, and Terpene profiles using independently evolved and co-evolved lines across two juices (9999 permutations).

	Effect	*df*	SS	MS	F_{pseudo}	R^2	P
Esters	Juice	1	0.37632	0.37632	6.5290	0.22104	0.0121
	Culture status	1	0.25674	0.25674	4.4543	0.15080	0.0334
	Interaction	1	0.03194	0.03194	0.5541	0.01876	0.5288
	Residuals	18	1.03750	0.05764		0.60940	
	Total	21	1.70250			1.00000	
Fatty acids	Juice	1	1.0273	1.02727	8.2687	0.29638	0.0001
	Culture status	1	0.1238	0.12376	0.9961	0.03571	0.3822
	Interaction	1	0.0788	0.07878	0.6341	0.02273	0.6884
	Residuals	18	2.2362	0.12424		0.64519	
	Total	21	3.4660			1.00000	
C6 compounds	Juice	1	0.58652	0.58652	99.038	0.78388	0.0001
	Culture status	1	0.02806	0.02806	4.738	0.03750	0.0309
	Interaction	1	0.02705	0.02705	4.567	0.03615	0.0320
	Residuals	18	0.10660	0.00592		0.14247	
	Total	21	0.74823			1.00000	
Higher alcohols	Juice	1	1.47854	1.47854	281.632	0.93292	0.0001
	Culture status	1	0.00574	0.00574	1.093	0.00362	0.2086
	Interaction	1	0.00607	0.00607	1.156	0.00383	0.3678
	Residuals	18	0.09450	0.00525		0.05963	
	Total	21	1.58484			1.00000	
Terpenes	Juice	1	0.47113	0.47113	24.7756	0.52730	0.0001
	Culture status	1	0.07879	0.07879	4.1434	0.08818	0.0203
	Interaction	1	0.00127	0.00127	0.0669	0.00142	0.9720
	Residuals	18	0.34229	0.01902		0.38309	
	Total	21	0.89348			1.00000	

1.2 Figures

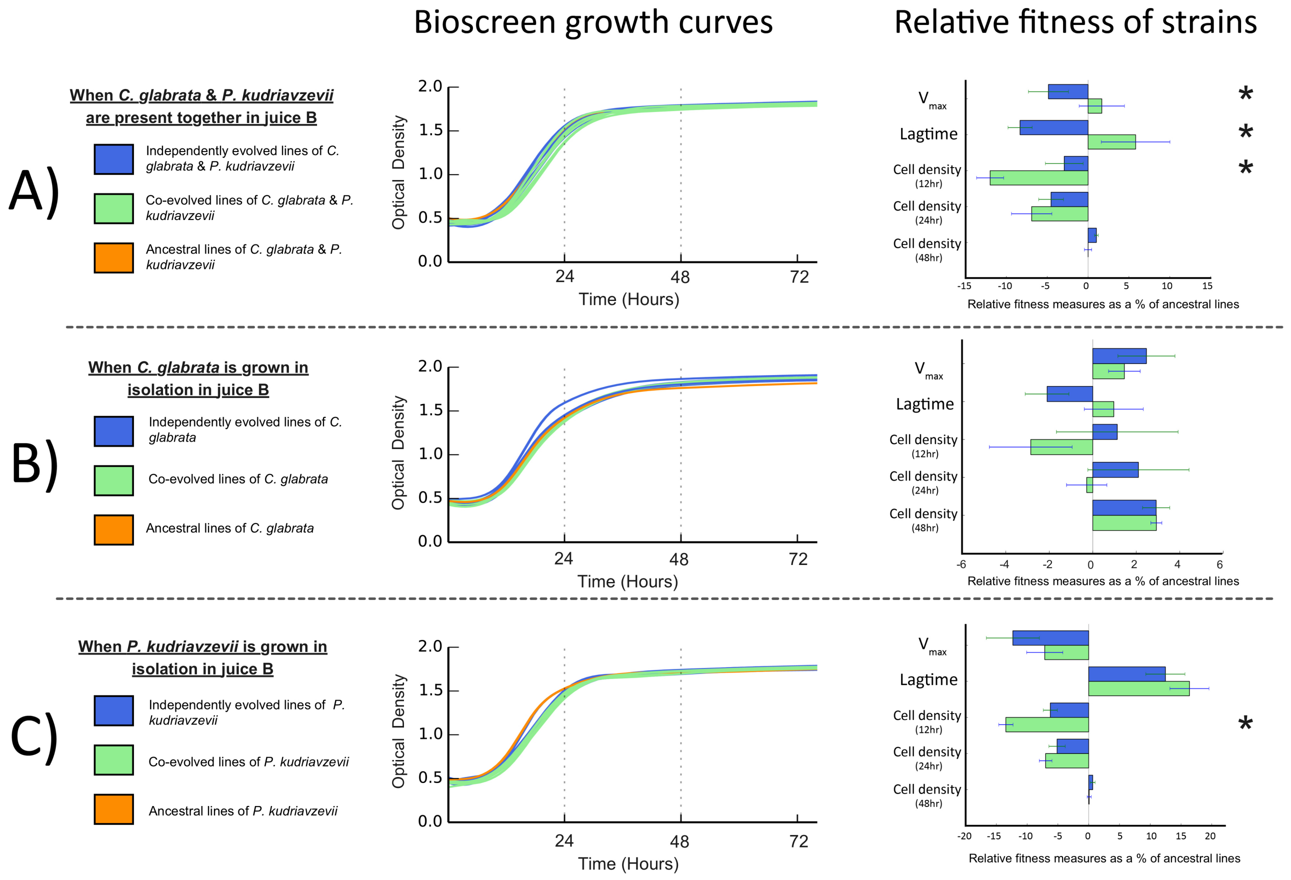

Figure S1: Bioscreen^{TM} growth curves and relative fitness measures in juice B of co-evolved, independently evolved, and ancestral strains of A) *C. glabrata* and *P. kudriavzevii* when grown together. B) *C. glabrata* when grown in isolation. C) *P. kudriavzevii* when grown in isolation. Relative fitness measures – V_{max}, Lagtime, and Cell densities – are expressed as the difference between evolved strains and the average ancestral strain expressed as a proportion of the average ancestral strain. Significant differences between co-evolved and independently evolved strains are denoted by '$*$'.

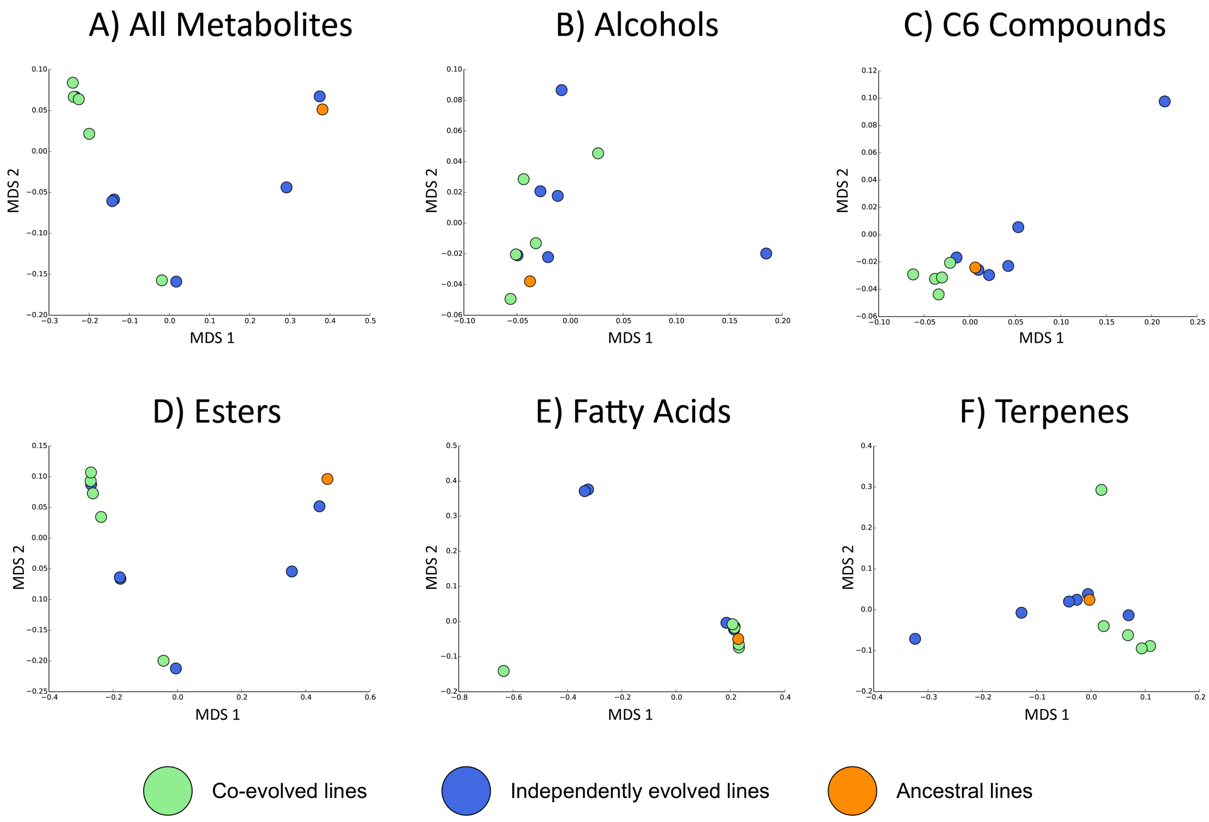

Figure S2: Jaccard dissimilarities of metabolite profiles – averaged across technical replicates – for A) All metabolites, B) Higher alcohols, C) C6 compounds, D) Esters, E) Fatty acids, F) Terpenes in Juice B.

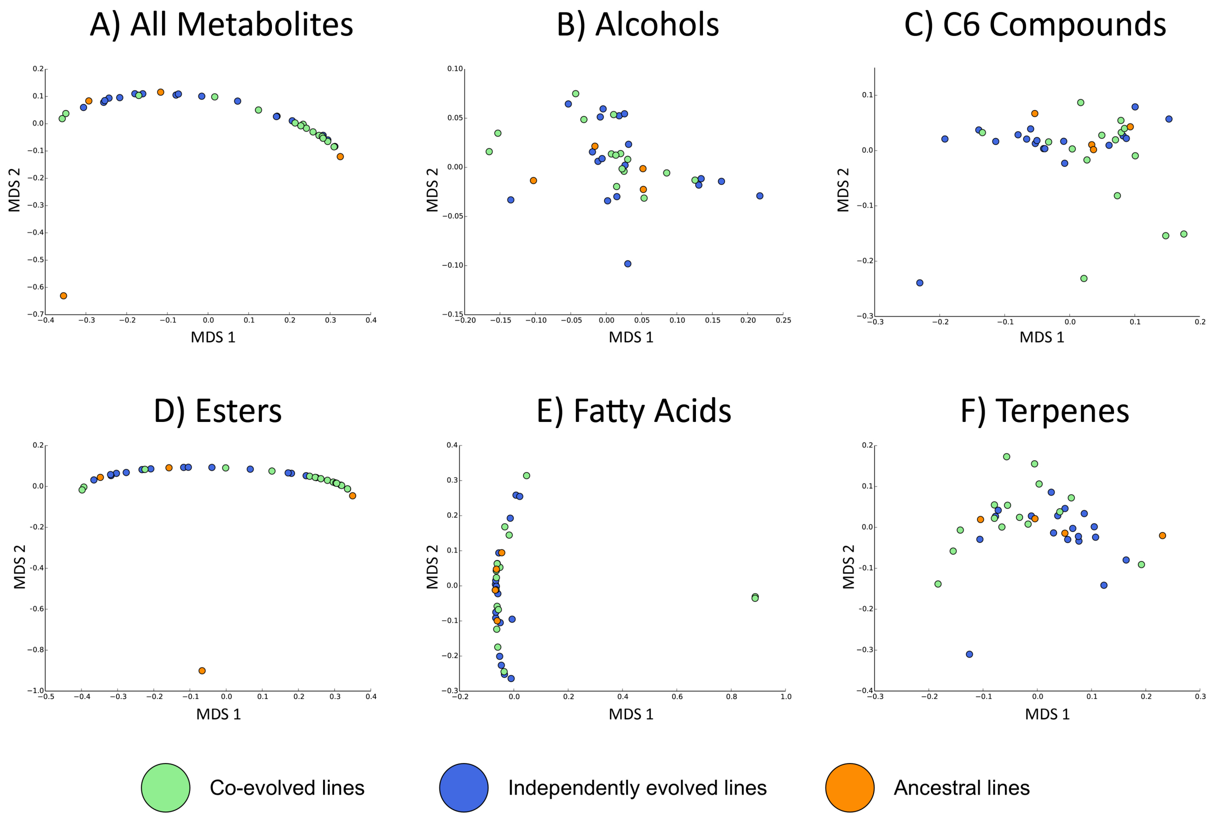

Figure S3: Jaccard dissimilarities of metabolite profiles of all experimental ferments – including all technical replicates – for A) All metabolites, B) Higher alcohols, C) C6 compounds, D) Esters, E) Fatty acids, F) Terpenes in Juice A.

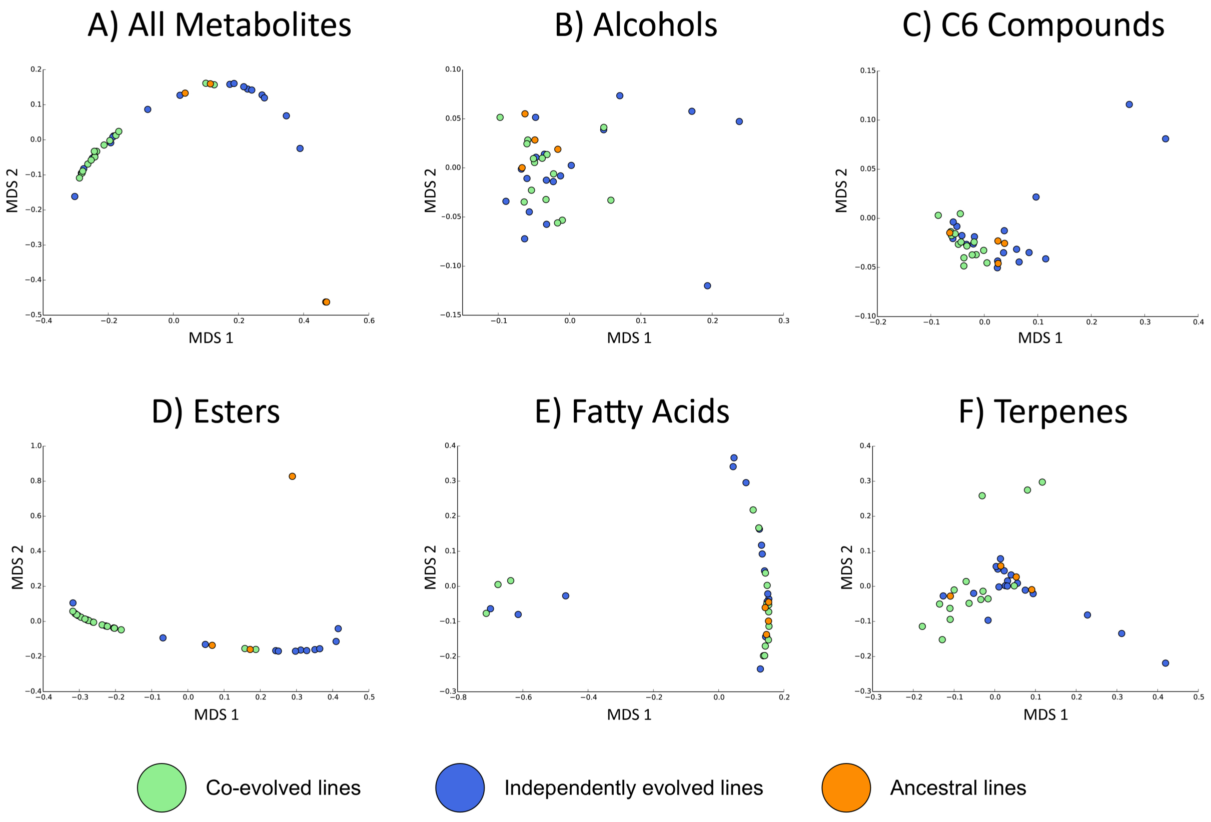

Figure S4: Jaccard dissimilarities of metabolite profiles of all experimental ferments – including all technical replicates – for A) All metabolites, B) Higher alcohols, C) C6 compounds, D) Esters, E) Fatty acids, F) Terpenes in Juice B.

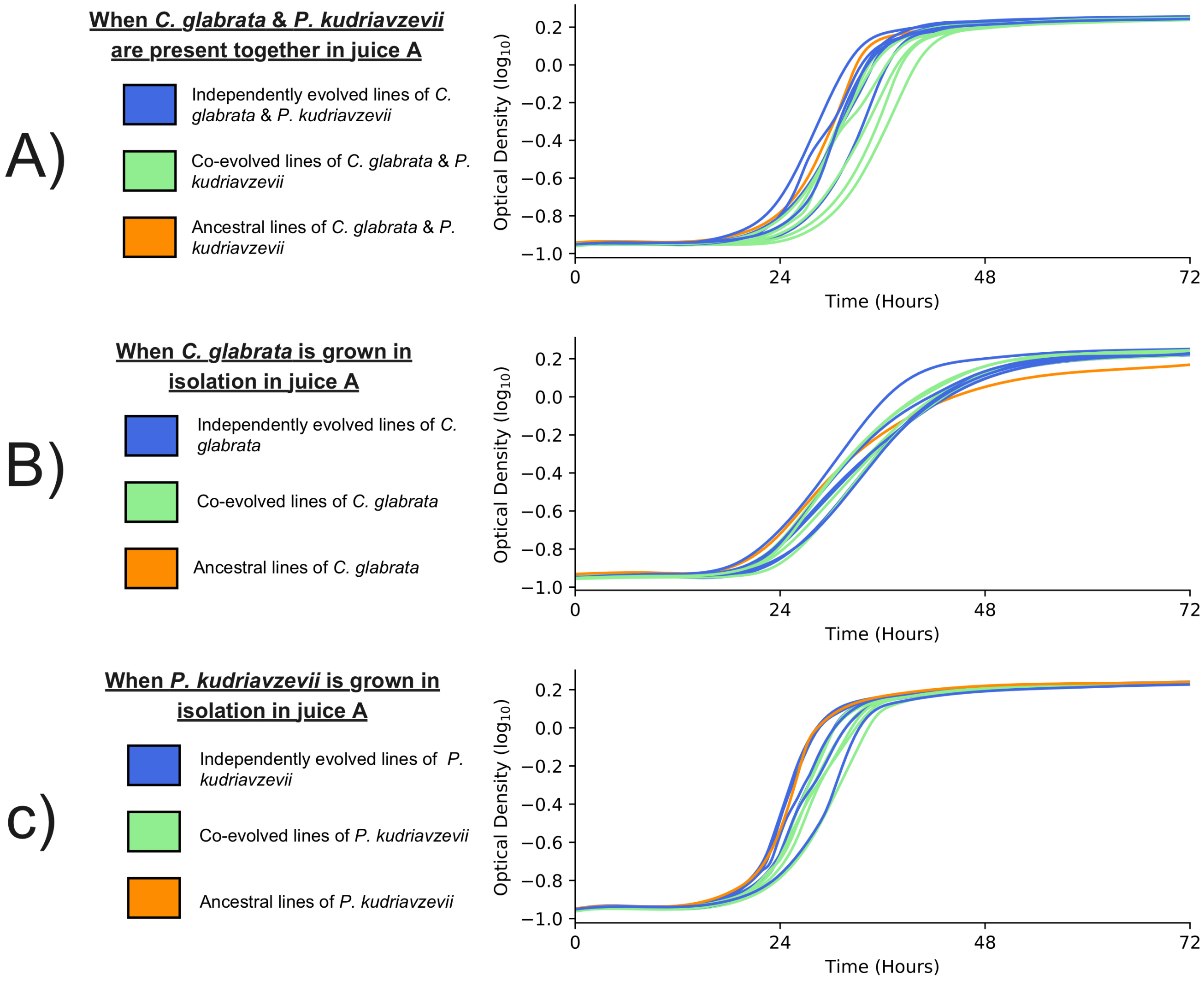

Figure S5: Log_{10} transformed optical densities of Bioscreen^{TM} growth curves in juice A of co-evolved, independently evolved, and ancestral strains of A) *C. glabrata* and *P. kudriavzevii* when grown together. B) *C. glabrata* when grown in isolation. C) *P. kudriavzevii* when grown in isolation.

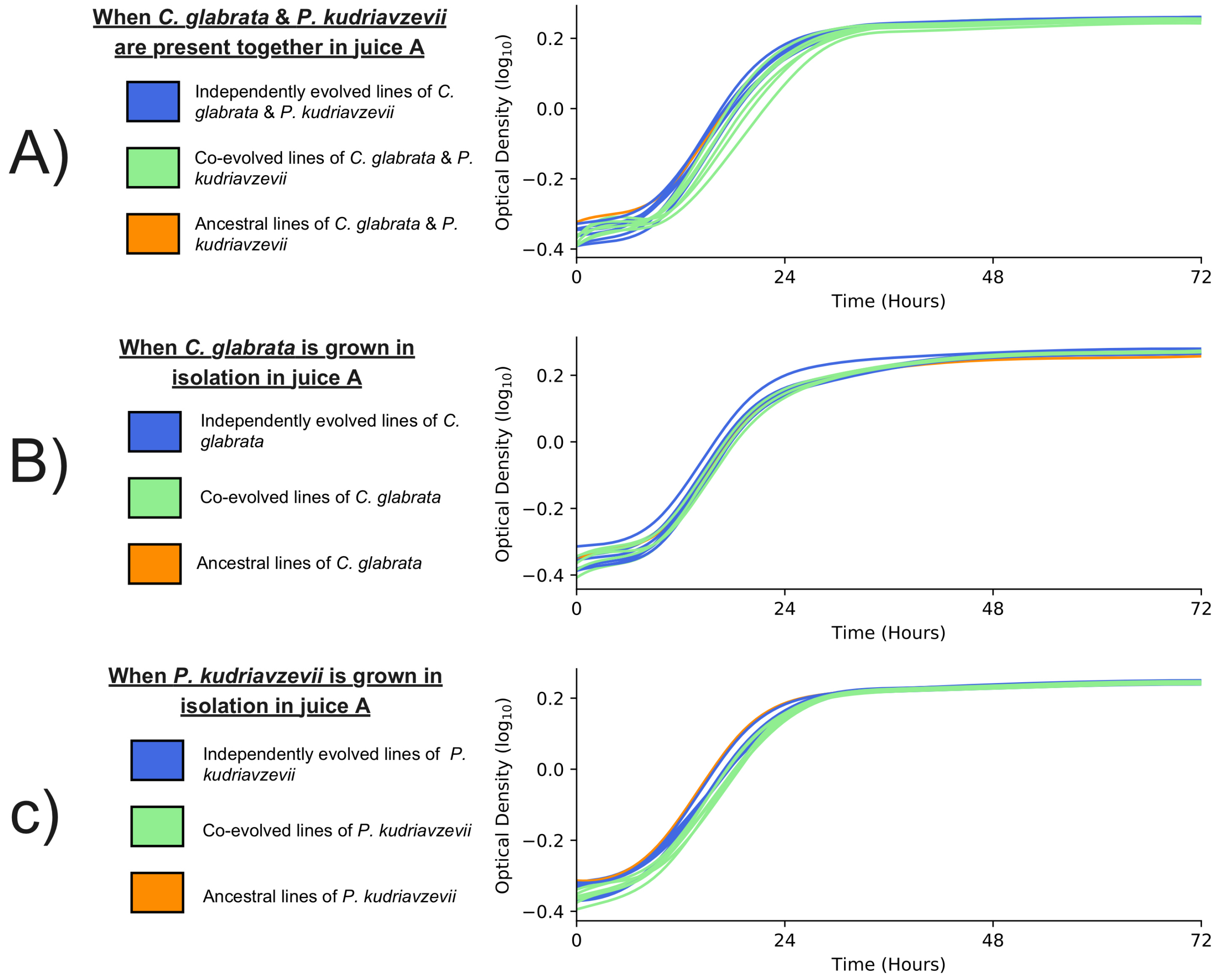

Figure S6: Log_{10} transformed optical densities of Bioscreen^{TM} growth curves in juice B of co-evolved, independently evolved, and ancestral strains of A) *C. glabrata* and *P. kudriavzevii* when grown together. B) *C. glabrata* when grown in isolation. C) *P. kudriavzevii* when grown in isolation.

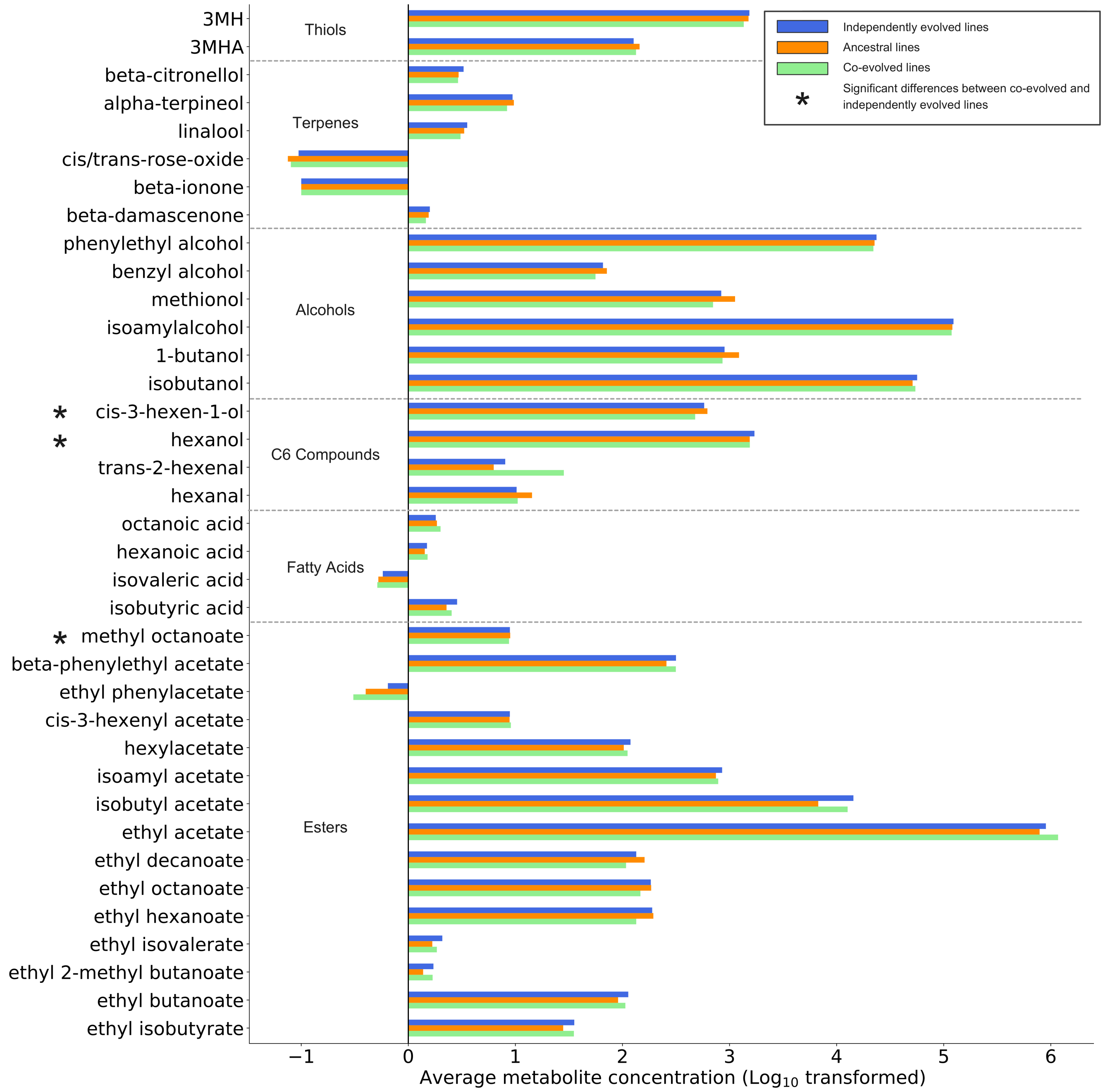

Figure S7: Log_{10} transformed metabolite concentrations of independently evolved, ancestral, and co-evolved lines that were inoculated in Juice A - the juice they were evolved in.
